# Supplementary material for: Usual prevention in unusual settings: A scoping review of place-based health interventions in public-facing businesses
Source: PLoS One. 2025 Jan 24;20(1):e0317815. doi: 10.1371/journal.pone.0317815 (PMC11760570; doi:10.1371/journal.pone.0317815)
Supplement: S1 Table — (DOCX) [file pone.0317815.s001.docx]

Supplemental Table 1. Keywords/operators/truncation used in databases for systematic review

| **Databases** | **Search Terms** |
| --- | --- |
| PubMed, Google Scholar, and APA PsycNet | ("public health" AND (barbershop OR laundromat OR "hair salon" OR "movie theater" OR "nail salon" OR mechanic OR "business setting" OR "unconventional setting" OR "community-based business" OR "non-traditional business" OR "service industry" OR "retail environment") AND (intervention OR education OR pharmacy OR healthcare OR "health service" OR "disease prevention" OR "health promotion" OR "health screening")) |
