# Supplementary material for: Usual prevention in unusual settings: A scoping review of place-based health interventions in public-facing businesses
Source: PLoS One. 2025 Jan 24;20(1):e0317815. doi: 10.1371/journal.pone.0317815 (PMC11760570; doi:10.1371/journal.pone.0317815)
Supplement: Attachment — Submitted filename: Response Letter.docx [file pone.0317815.s003.docx]

October 15, 2024

Dear Dr. Chekuri and Reviewers

Thank you for the opportunity to revise and resubmit our article entitled “Usual prevention in unusual settings: A scoping review of place-based health interventions in public-facing businesses” (PONE-D-24-30536). We appreciate the Reviewers’ constructive comments and have worked to address each of them, which we detail below. We believe the review has been strengthened in the process and hope you can reconsider this work for publication in PLOS ONE. Thank you greatly for your time.

**For Reviewer #1:**

1. “The study fails to clarify how this scoping review builds upon a recent umbrella review by McGowan et al (2021). In addition, this article’s main finding of use of barbershops and beauty salons for public health outreach, research, and health intervention in racial and ethnic minority populations is already well established. For example, W. Kong and E. Saunders published in this context about hypertension in 1989. Additional synthesis of data by health issue, geographic region, strength of evidence, etc. would be helpful to the reader. For example, an evidence map may be relevant.”

**Response #1: We appreciate the reviewer’s suggestion to clarify how this review builds on existing literature, such as the recent umbrella review by McGowan et al. (2021). In response, we have expanded the introduction to clarify that, unlike prior reviews that largely focus on interventions in more conventional settings, our scoping review examines preventive health interventions in a broader array of unconventional business settings. We have revised the text on pages 2-3 to emphasize this distinction and highlight our review’s unique focus on public-facing venues outside of commonly studied sites such as grocery stores and pharmacies.**

2. “I have concerns about the level of sufficient detail to determine whether the scoping review was performed with rigor. Increased transparency in the methods is essential. The authors are off to a good start by using the PRISMA checklist for reporting. However, several key areas are not included or are included with insufficient detail. There was only one reviewer and yet no information about attempts to reduce bias. They should thoroughly address failure to implement best practices from Mak and Thomas (2022). For example, the review protocol should be registered and the management software named. They should describe methods used to synthesize results. They should describe the process of extraction, calibration, stakeholder consultation, and beyond. The lack of assessment on risk of bias or analysis of strength of evidence should be explored as described in the PRISMA checklist.”

**Response #2: Thank you for your feedback on ensuring sufficient rigor in our methods. We have revised the manuscript to clarify that two reviewers participated in all stages of the review, including with one reviewer leading study screening, selection, and data extraction and checked by the second reviewer with corrections made when discrepancies were found. We provided additional detail on our calibration process, where both reviewers independently extracted data from a sample of studies to ensure consistency. We also clarified our management approach, noting the use of Excel for data collection and Zotero for citation management. While the review protocol was not pre-registered, we aligned our methods with PRISMA guidelines for scoping reviews. We acknowledged the lack of formal stakeholder consultation as a limitation. Additionally, we described our results synthesis by categorizing studies according to intervention type, health condition, and setting. Finally, we assessed the methodological quality of each study using the NIH Study Quality Assessment Tools to provide insights into the strength of the evidence presented. These changes were implemented on pages 3-6 and 18 to enhance the transparency and rigor of our scoping review.**

3. “As part of the discussion, in exploring these findings, the unintended consequences (ie. stigma) should be noted in conducting any health intervention in a public space. Experience with community advisory boards helps tailor interventions with cultural competence. In addition, greater discussion of the limitations and gaps is needed.”

**Response #3: Thank you for your valuable feedback regarding the need to address stigma in health interventions. We have revised the manuscript to include this important aspect. On page 17, we now discuss how health interventions in public settings may lead to stigma, potentially affecting participant engagement. We highlight the importance of understanding these dynamics to ensure interventions are culturally competent and effectively tailored to the communities served. This addition strengthens our discussion and emphasizes the need to consider unintended consequences in place-based health interventions.**

4. “Figure 1 is very challenging to read despite zooming in. Make the text larger and increase the quality of the image. Additional details to clarify the process should be included.”

**Response #4:** We have now uploaded an original image for Figure 1 which should be higher resolution.

5. “’Black’ should be capitalized consistently throughout when referring to groups in racial, cultural, or ethnic terms.”

**Response #5: Thank you for your valuable feedback regarding the capitalization of "Black" in our manuscript. We have revised the text to ensure that "Black" is now consistently capitalized throughout when referring to groups in racial, cultural, or ethnic terms. We appreciate your attention to this detail, as it enhances the accuracy and professionalism of our writing.**

6. “Justification is needed on why only peer reviewed articles were included and what impact that could have on the findings.”

**Response #6: Thank you for your insightful comment regarding the inclusion of only peer-reviewed articles in our review. We chose to limit our analysis to peer-reviewed studies to ensure the rigor and credibility of the research findings. Peer review serves as a quality control mechanism that helps validate the methodology and results of a study, thereby enhancing the reliability of the evidence presented. By focusing on peer-reviewed literature, we aimed to provide a more robust synthesis of place-based health interventions, ensuring that the interventions evaluated have undergone critical scrutiny by experts in the field. We acknowledge that this choice may limit the scope of included studies and could exclude valuable insights from non-peer-reviewed literature. However, we believe that prioritizing peer-reviewed research contributes to the overall strength and validity of our review's conclusions. We now provide a justification in the Methods section.**

7. “The full syntax of keywords and strategies used in the initial searches should be available as supplemental materials, not available upon request from the authors.”

**Response #7: We now provide a full list of syntax used as an Appendix item.**

**For Reviewer #2:**

**Abstract**

1. “Please provide a clearly defined and focused objective. The current version is too broad, making it difficult for the reader to gauge the expectations and scope of the review. I recommend using the objective stated in the Introduction for consistency and clarity.”

**Response #1: Thank you for your insightful comment regarding the need for a clearer and more focused objective. In response, we have revised the objective in the abstract to better articulate the scope and expectations of the review. The objective now clearly states the aim of summarizing the peer-reviewed literature on place-based health interventions in unconventional public-facing business settings, specifically highlighting the target populations and health conditions addressed. These changes can be found on page 1 of the revised manuscript.**

2. “Additionally, a clarification is needed for the statement: “While there have been 9 randomized trials conducted across health conditions, they have been limited to a few select settings with little measurement of objective health outcomes indicating a need for more rigorously designed studies.” It is unclear whether the concern is the limited rigor of the randomized trials or their restriction to select settings with inadequate focus on objective health outcomes. I will suggest that authors address this ambiguity for a clearer interpretation.”

**Response #2: Thank you for your insightful feedback regarding the statement on the randomized trials. To address the ambiguity, we have revised the text for clarity. The updated sentence now explicitly distinguishes the two concerns regarding the randomized trials: their restriction to select settings and the limited rigor in their design, particularly concerning the measurement of objective health outcomes. The revised statement now more simply and clearly reads: “While there have been 9 randomized trials conducted across various health conditions, these trials are limited in two** **key ways: first, they focus on a few select settings and lack of objective health out. This highlights the need for more rigorously designed studies in diverse settings that can effectively evaluate the impact of place-based health interventions.” These changes were made on page 1. We appreciate your guidance in enhancing the clarity of our manuscript.**

**Introduction:**

3. “The statement, “However, these interventions were mostly focused on altering the ‘place’ of people as the intervention rather than focusing on delivering usual interventions in unusual places or settings,” requires expansion. It is not immediately apparent why altering the "place" of people is less favorable compared to delivering interventions in unconventional settings. Further explanation of these approaches and their respective merits or shortcomings would add depth to the argument.”

**Response #3: We appreciate your feedback regarding the need for expansion on the statement concerning the focus on altering the "place" of people versus delivering usual interventions in unconventional settings. In response, we have clarified this point in the paragraph by emphasizing that altering the “place” of people can disconnect individuals from their familiar community contexts. We argue that delivering traditional health interventions in unconventional settings—such as salons and barbershops—preserves the familiarity and trust these spaces offer, which can lead to better engagement and participation from target populations. This addition enriches the discussion by providing a clearer rationale for why the context of the intervention matters. These changes were made on page 2.**

4. “The example cited “For example, there have been a number of studies that have developed health promotion interventions in salons and barbershops to reach African American communities and a systematic review has summarized this research [3]” is a good one, but it feels disconnected from the preceding sentences. I will suggest re-writing the paragraph to provide a more coherent narrative, clearly linking examples to the argument.”

**Response #4: Thank you for your feedback regarding the coherence of the example within the paragraph. We have revised the paragraph to create a clearer and more cohesive narrative that better links the example to the overarching argument. Specifically, we clarified how altering the "place" of people can disconnect them from familiar community settings, in contrast to delivering interventions in unconventional but trusted spaces like salons and barbershops. This revision illustrates how using such settings can enhance engagement and accessibility for target populations. These changes were made to improve the flow and strengthen the argument within the paragraph on pages 2-3.**

5. “There is also a lack of an explicit explanation of how this review fills a gap in the literature. Currently, the significance of focusing on "unusual business settings" for health interventions is not adequately emphasized. Further explanation on why these settings are essential for addressing health disparities or improving outreach would strengthen the introduction. The objective should also address the "so what?" question to clarify the overall goal of the review.”

**Response #5: Thank you for highlighting the need for a clearer explanation of this review’s significance. In response, we have revised the introduction to explicitly state how focusing on unconventional business settings for health interventions addresses a gap in the literature. We emphasize that these settings—such as salons, barbershops, and other non-traditional venues—provide a unique opportunity to reach underserved populations in familiar, trusted spaces, potentially enhancing engagement and access to health resources. We have also clarified the objective to highlight how these unconventional settings can contribute to addressing health disparities and extending outreach. These revisions were made to ensure that the purpose and importance of the review are clear, providing a stronger rationale for its focus.**

**Methods:**

6. “First, the manuscript states that "four major search databases were used: PubMed, Google Scholar, and APA PsycNet," but this lists only three databases.”

**Response #6: Thank you for catching this inconsistency. We have corrected the text to reflect that three major search databases were used (PubMed, Google Scholar, and APA PsycNet) instead of four. This change has been made on page 4.**

7. “Also, the rationale behind limiting the search to studies from 1995-2023 is not provided. An explanation for this temporal restriction is needed to justify its relevance to the review.”

**Response #7: Thank you for your comment. We have clarified in the manuscript that while the search range in each database was set from 1960 to the present, the studies that met our inclusion criteria were published between 1995 and 2023. This revision has been made on page 4.**

8. “The search syntax appears overly simplistic. I recommend following established search strategies from previous reviews, such as those by McGowan et al. (2021) and Palmer et al. (2021), which may provide more robust search terms. In addition, the search seems to focus exclusively on barbershops, laundromats, hair salons, movie theaters, and nail salons, but there is no explanation for excluding other business settings that might serve as intervention sites. Expanding this scope, or at least justifying the exclusion of other settings, would improve the comprehensiveness of the review.”

**Response #8: Thank you for this feedback. We have now included the exact search terms and syntax used in our search, which included the strategies suggested. We focused on sites, such as barbershops, laundromats, hair salons, movie theaters, and nail salons—that met our definition of "unconventional" business settings where healthcare interventions had not usually be considered historically. These settings were selected based on their established role in the community and their alignment with our review’s objective of identifying less traditional sites for health interventions. Expanding to additional settings was beyond the scope of this review; however, we agree that exploring a broader range of business types could be valuable for future research.**

9. “It is best practice to include both inclusion and exclusion criteria explicitly, which are missing in this section. Additionally, since the review appears to be conducted by a single author, this raises concerns about potential bias. I recommend that co-authors participate in the data extraction process and that at least two reviewers are involved to verify extracted data and ensure consistency, which will strengthen the rigor of the review.”

**Response #9: Thanks for this important point. We have now clarified both inclusion and exclusion criteria explicitly. In short, inclusion criteria were “studies conducted in the United States, provided health interventions (e.g., education, disease screening, connection with healthcare providers, pharmacy services) to clients in one or more business settings for one or more diseases or conditions, were written in English, and were peer-reviewed.” And exclusion criteria were “studies that were not peer-reviewed, not published in English-written journals, or did not report study of a health intervention in a business setting.Results:**

10. “The current organization of the results does not align well with the review’s objectives. I recommend thematizing the findings based on the various types of preventive health interventions rather than organizing them by health conditions. This would allow for a more structured discussion of intervention methods, such as screenings for hypertension, HIV, or diabetes, while also categorizing them into primary, secondary, and tertiary prevention levels.”

**Response #10: Thank you for your thoughtful comment on the organization of the results. We appreciate your suggestion to thematize findings based on types of preventive health interventions. We tried to reorganize them as suggested but found the organization by health conditions best aligns with the objectives of the review and is easier for readers to synthesize the literature. For example, structuring Tables 1, 2, and 3 by condition—CVD, HIV, and Diabetes; Cancer; and other conditions—enables a focused examination of interventions within each health context, which we believe is beneficial for researchers and practitioners. By grouping studies this way, we allow readers to understand specific intervention effects by condition and geographic setting, which might be less apparent in a purely thematic organization. We will, however, emphasize intervention types within each table to further highlight this information. We hope you agree with this approach, thank you.**

11. “For these sentences, “Table 1 contained studies providing interventions for cardiovascular health conditions, HIV, and diabetes. Table 2 contained studies providing interventions for different cancers, and Table 3 contained studies providing interventions for all other conditions not falling.”, Please cite the studies that belong to each of these categories. For instance, when discussing cardiovascular interventions, specific studies should be cited for clarity.”

**Response #11: Thank you for your suggestion to cite specific studies associated with each category in Tables 1, 2, and 3. We have now incorporated these citations to clarify which studies fall under each health condition category, such as cardiovascular interventions, cancer, and other conditions.**

12. “The sentence, "These studies included screening programs, cross-sectional studies, longitudinal studies, descriptive studies, feasibility studies, a randomized controlled trial, pilot studies, cohort studies, and program evaluations," seems unclear in its inclusion of screening programs. Is there a specific reason screening programs are listed alongside study designs? Clarifying this distinction would improve clarity.”

**Response #12: Thank you for pointing out the potential confusion with listing "screening programs" alongside study designs. To enhance clarity, we have reclassified the studies and removed "screening programs" as a study design. This adjustment aligns the classifications more precisely with the study designs used in the review.**

13. “Including more commentary on intervention outcomes, particularly which intervention methods were most effective, would add depth to the analysis… Adding more commentary on intervention outcomes, particularly on which methods were the most effective, would enrich the analysis and provide readers with a clearer sense of the practical impact of these interventions…More detail on the effectiveness of the interventions is needed, especially in terms of measurable health outcomes. For instance, while the review notes positive participant responses, including data on health improvements or behavior changes (e.g., increases in screenings or follow-up rates) would strengthen the results section.”

**Response #13: Thank you for suggesting additional commentary on intervention outcomes and effectiveness. Since the primary aim of this review was to assess the types of health interventions conducted for various conditions within unconventional business settings, rather than to evaluate intervention effectiveness, we focused our efforts on describing these elements as a way to map the landscape of where the literature is at this stage. However, we have worked to include more notes about outcomes (when available which was not always the case) and now include our ratings of study quality as well. One of our findings is that the lack of rigor of studies makes it hard to compare health interventions across diverse settings, hopefully paving the path for future comparative effectiveness research.**

14. “The tables are well-structured, but adding a column that summarizes the limitations of each study—such as small sample sizes or the observational nature of some interventions—would be helpful, especially since the authors are interested in the rigor of study designs.”

**Response #14: Thank you for suggesting the addition of a column to summarize study limitations. We have not spent efforts to assess the quality of each study using the NIH Study Quality Assessment Tools, which considers various limitations, such as sample size and study design rigor, where applicable. These quality assessments are reflected in our quality rating for each study, as seen in the tables.**

**Discussion:**

15. “The discussion section should demonstrate stronger alignment with the review's objective. For instance, the statement "we reviewed 43 studies of place-based health interventions" should clearly connect to the objective, which is to assess interventions delivered in non-traditional settings.”

**Response #15: Thank you for your comment on strengthening the alignment between the discussion and the review's objective. We have clarified in the discussion section that the review specifically examines interventions delivered in non-traditional or unconventional business settings. This addition reinforces the unique focus of our review and its objective to assess health interventions outside of standard healthcare environments.**

16. “Additionally, the specific health condition with the greatest number of rigorous research designs (e.g. randomized controlled trials, cluster randomized trials) was for hypertension (3 studies).” I do not agree with this statement. The claim that randomized controlled trials are more rigorous than other study designs is problematic. The rigor of a study design depends on its alignment with the research question. Cross-sectional studies can be as rigorous as randomized controlled trials if they are appropriately suited to the research aims.”

**Response #16: Thank you for your thoughtful comment. We appreciate your point regarding the rigor of different study designs and it is dependent on study aims. We have the challenge of trying to use some general metric and so we rely on the evidence hierarchy with randomized controlled trials as representing the most rigorous designs. We now revise our wording to simply state that the specific health condition with the greater number of controlled trials was hypertension.**

17. ““Further study is needed to expand beyond these settings to determine whether other public-facing business settings (e.g., grocery stores, banks, movie theatres, malls) are also effective and acceptable places for health interventions.” Did any of the reviewed studies focus on these settings or is there a reason why authors did not review articles from these settings?”

**Response #17: Thank you for your comment. We did find studies that were conducted in grocery stores, but we considered them to be more conventional business settings for healthcare (e.g., pharmacies, optometrists, etc.), which did not align with the focus of our review on non-traditional business settings. As for other public-facing business settings like banks, movie theaters, and malls, no studies we did not mention in our review met our full inclusion criteria. Given the lack of studies in these areas, we reframed the discussion to highlight the need for future research to explore the effectiveness and acceptability of health interventions in these settings.**

18. “The sentence, "Although we counted 7 randomized trials, including several cluster randomized trials, they offered different types of interventions and targeted different health conditions, making synthesis challenging," is unclear. Could the authors clarify whether they attempted to synthesize information from these studies? If not, what approach did they take to handle these trials?”

**Response #18: Thank you for your comment. We did not attempt to synthesize the results from the 7 randomized trials due to their heterogeneity in interventions and health conditions. We chose to summarize each study individually, which allowed us to capture their diverse nature. We recognize that a more focused synthesis could be useful in future research on specific interventions or health conditions. These changes have been made on pages 17 and 18.**

19. “Many of these trials appeared to have design flaws, potentially compromising internal validity due to factors such as confounding variables, inadequate sample sizes, limited follow-up, or differential attrition between groups." Since the authors are concerned with study design rigor, it would be beneficial to evaluate the quality of each trial using established guidelines.”

**Response #19: Thank you for your comment. We agree that evaluating study quality is important. To address this, we conducted quality assessments for each study using the NIH Study Quality Assessment Tools. These assessments helped us evaluate the rigor of the study designs, accounting for factors such as internal validity and other potential design flaws.**

20. “The statement "Almost all studies relied on subjective outcome measures and did not measure..." is incomplete and not discussed in the results section. The authors should include this in the results if it is an important observation.”

**Response #20: Thank you for your comment. We appreciate your observation. However, we would like to note that our results section does include a discussion of the outcome measures used in the studies, including the reliance on subjective measures. The issue of objective versus subjective outcomes is highlighted both in the results and the discussion to emphasize the limitations in study design.**

21. “Given the range of studies, we are also limited in specificity in drawing conclusions” Authors should please clarify what this statement means.”

**Response #21: Thank you for your comment. The statement refers to the variability in study designs, interventions, and health conditions across the studies reviewed, which made it challenging to draw specific, detailed conclusions. Given that the studies varied widely in terms of methods and outcomes, synthesizing precise conclusions across all studies was not feasible. This limitation is a result of the scoping review's broad approach to capturing a diverse range of interventions and settings, which inherently limits the specificity of the findings.**

22. “You only included studies from the United States, but there may be innovative place-based interventions in other countries" requires justification in the introduction. Why was the focus restricted to U.S.-based studies?”

**Response #22: Thank you for your comment. The focus on U.S.-based studies was primarily due to the aim of this review to assess place-based health interventions within the unique context of the U.S. healthcare system and business environments. Given that the review targeted unconventional business settings, such as barbershops and beauty salons, which are especially prevalent in the U.S., it was necessary to restrict the scope to studies conducted in the U.S. This allowed for a more focused analysis of health interventions within these specific settings. But we would greatly value examination of international studies that would certainly generate valuable insight so we now state this a study limitation of our review and an opportunity for future research, thank you.**

**General:**

23. “While the review highlights innovative methods for reaching underserved populations in barbershops and beauty salons, the current structure of the results may not fully support this claim. Reorganizing the results by types of place-based health intervention first, followed by specific interventions, and then health conditions, would provide a clearer framework for discussion.”

**Response #23: Thank you for your suggestion. While we appreciate the recommendation to reorganize the results, we chose to structure the review by health conditions to better align with the objective of assessing the interventions for specific chronic health conditions in non-traditional business settings. We believe that this structure provides a clearer understanding of the types of interventions associated with each condition and allows readers to see how these interventions are distributed across various health issues. However, we acknowledge the merit in organizing by intervention type and will consider this suggestion for future iterations or research in this area to further enhance clarity and structure.**

24. “A more detailed exploration of the barriers encountered in implementing health interventions in these settings should be included in the discussion. Challenges related to business owners’ willingness to participate, or logistical issues should be addressed.”

**Response #24: Thank you for the suggestion. We recognize that barriers such as business owners’ willingness to participate and logistical challenges are important considerations in the implementation of health interventions. However, as the primary objective of this review was to assess the types of interventions offered in unconventional business settings, exploring these barriers was outside the scope of the study. That said, we acknowledge the value of understanding these challenges in future research to enhance the effectiveness and scalability of such interventions. While we did not address these barriers directly, we will consider mentioning them briefly in the discussion to highlight the complexities involved in implementing place-based health interventions.**

25. “Expanding the discussion to include policy implications for promoting place-based health interventions, particularly in underserved communities, would add valuable context to the findings.”

**Response #25: Thank you for the suggestion. While we agree that policy implications are an important aspect of promoting place-based health interventions, particularly in underserved communities, this topic falls outside the scope of the current study. Our primary focus was to review the types of health interventions delivered in unconventional public business settings. However, we appreciate the importance of policy discussions in the broader context of implementing these interventions and will consider it as a potential direction for future research or discussion.**

26. “Additionally, reflecting on how the review's findings could guide future research on health disparities would strengthen the overall contribution of the paper.”

**Response #26: Thank you for the suggestion. We have already addressed how the findings of this review could guide future research on health disparities within the discussion section. Specifically, we highlight the need for more rigorous and varied studies to better understand the effectiveness of place-based interventions, particularly in reaching underserved populations. We appreciate the emphasis on future research directions and believe our existing comments in the discussion align with this suggestion.**
