# Supplementary material for: Usual prevention in unusual settings: A scoping review of place-based health interventions in public-facing businesses
Source: PLoS One. 2025 Jan 24;20(1):e0317815. doi: 10.1371/journal.pone.0317815 (PMC11760570; doi:10.1371/journal.pone.0317815)
Supplement: Attachment — Submitted filename: Editor comments 12-15-24.docx [file pone.0317815.s004.docx]

Thank you for your scholarly contribution. I'd also like to thank the authors for choosing PLOS ONE to publish your findings from this study. Comments from reviewers and my suggested corrections are provided below. Please review these comments and suggestions and I suggest addressing them and resubmit your manuscript. Your timely response would help this study be published and will make it accessible to interested readers across the world. I look forward to reviewing your revised manuscript. I wish you good luck with your future endeavors.

Please consider adding: “Health care interventions”, “public settings”, “Place-based interventions” to the keyword list.

Page two: consider revising the phrase: "creating bike lanes to increase exercise" to "creating bike lanes to ~~increase~~ **facilitate** exercise"

While fig 1 shows studies included in the review at "42". Your in-text description (Abstract and also on page 4) pegs these studies at "43". Please clarify.

On Page 4: "After a secondary review (NM), four more studies were removed". Fig 1 illustrates “5 studies were removed”. Please clarify.

On Page 8: Please consider revising this phrase: "a medication highly effective at reduced the risk of getting HIV" to "a medication highly effective at ~~reduced~~ **reducing** the risk of getting HIV)

On page 9: Please consider revising this sentence: "The Shape Up! intervention led to significant increased consistent condom use in the postintervention period." to "The Shape Up! intervention led to ~~significant~~ **a significantly** increased consistent condom use in the postintervention period."

Likewise, "These three notable studies together suggest place-based interventions can lead to decreased risky behavior associated HIV " to "These three notable studies together suggest place-based interventions can lead to decreased risky behavior associated HIV **transmission**".

Likewise, "and incorporated diabetes education and preventions strategies" to "and incorporated diabetes education and ~~preventions~~ **prevention** strategies"

Likewise, On page 12: "Through the 10,306 kiosk used over a four year period...." to "Through the 10,306 ~~kiosk~~ **kiosks** used over a four year period..."

Likewise, On page 12, "Samples sizes of each of these studies ranged from 40-1,552 participants." to "~~Samples~~ **Sample** sizes of each of these studies ranged from 40-1,552 participants."

Likewise, on page 13, "Three studies provided colorectal cancer interventions in barbershops as a longitudinal study, a qualitative analysis, and a randomized controlled trial [7,28,30]." to "Three studies provided colorectal cancer interventions in barbershops as a longitudinal study, a qualitative analysis, and a randomized controlled trial **respectively** [7,28,30]."

Likewise, on page 14, "Another interesting study investigated the effectiveness of using beauticians to educate black female clients about stoke warning signs and risk factors [46,49]." to "Another interesting study investigated the effectiveness of using beauticians to educate black female clients about ~~stoke~~ **stroke** warning signs and risk factors [46,49]."

Page 15, second sentence: "stroke warming signs" to "stroke ~~warming~~ **warning** signs"

Page 15: Discussion section first two sentences: "found a number of studies" to "found **that** a number of studies"

Page 15: "select business settings" to "~~select~~ **selected** business settings"

Page 16: "Among the 43 studies reviewed, there was a lack of rigorously designed studies." "Among the 43 studies reviewed, there was a **general** lack of ~~rigorously~~ **rigor in the** designed studies."

Page 31: "Unkown" to "Unknown"
