# Supplementary material for: Usual prevention in unusual settings: A scoping review of place-based health interventions in public-facing businesses
Source: PLoS One. 2025 Jan 24;20(1):e0317815. doi: 10.1371/journal.pone.0317815 (PMC11760570; doi:10.1371/journal.pone.0317815)
Supplement: Attachment — Submitted filename: Response Letter 12.17.24.docx [file pone.0317815.s005.docx]

December 17, 2024

Dear Dr. Chekuri and Reviewers

Thank you for the opportunity to revise and resubmit our article entitled “Usual prevention in unusual settings: A scoping review of place-based health interventions in public-facing businesses” (PONE-D-24-30536R1). We appreciate the Editor’s careful reading and suggested edits along with each of the Reviewers’ positive and encouraging comments.

In this revised submission, we have now made all of the Editor’s suggested edits throughout the manuscript. For Reviewer #2, we have clarified the two sentences (bottom of page 2 and top of page 3) that there have been a few novel studies of interventions in unconventional settings like barbershops, but the majority of studies and the work in real-world settings continue to be in traditional settings like pharmacies and community health centers. We hope these revisions have improved the manuscript.

We greatly appreciate the opportunity to resubmit our work and hope this revised manuscript can be considered for publication. Thank you for your time.
