# Supplementary material for: Usual prevention in unusual settings: A scoping review of place-based health interventions in public-facing businesses
Source: PLoS One. 2025 Jan 24;20(1):e0317815. doi: 10.1371/journal.pone.0317815 (PMC11760570; doi:10.1371/journal.pone.0317815)
Supplement: Attachment — Submitted filename: Academic Editor comments 12-26-24.docx [file pone.0317815.s006.docx]

Thank you for your revisions. I am tentatively accepting this manuscript for publication pending some minor recommended changes as described below. Please review these comments and I suggest addressing them and resubmitting your manuscript. Your timely response would help this study be published and will make it accessible to interested readers across the world. I look forward to reviewing your revised manuscript. I wish you good luck with your future endeavors.

In the abstract section on page 1, I noticed an error in the following statement of yours. “The most common unconventional public-facing business settings for health interventions included barbershops and beauty salons (42 studies)…..”. Looks like there was one study (Peddecord et al., 2008) that described “Movie theaters” as their only setting (see table 3, page 36 for reference). This study did not describe “barbershops and beauty salons”. Hence, I'd recommend changing the number “42” above to “41”. Moreover, there were several studies that described either barber shops or beauty salons as their setting, and not both. Hence please revise this phrase “barbershops and beauty salons (42 studies)” to “barbershops ~~and~~ **or** beauty salons (41 studies)”.

In the abstract section on page 1, please revise “Notably, 34 (79%) of the studies targeted Black populations.” to “Notably, 34 (~~79~~ **81**%) of the studies targeted Black populations.” Since you corrected the number of studies that were reviewed to 42, the percentage needs a correction as well.

On page 2, please consider revising "Place-based interventions is one approach to do that to reach people “where they are.”" to "Place-based interventions is one approach ~~to do that~~ to reach people “where they are.”"

On top of page 3, there is a typo (“occur”) and extra space between words (“…literature, continue…”). Please correct this.

Page 3, second paragraph: Please consider revising the phrase "....interventions delivered less conventional...." to ".....interventions delivered **at** less conventional....."

Page 3, second paragraph: The following sentence is too complex and difficult to comprehend. Please revise and simplify. "Such a review would help public health practitioners, researchers, and business owners understand the types of public-facing business settings in which it is possible to deliver these interventions, which health conditions are targeted, what outcomes have resulted, and what lessons might be learned in reaching various underserved and minority populations in these settings."

On page 5, "Review process" first sentence: please delete the word "reviewers" from the sentence.

On page 7 last paragraph: Please revise this phrase “….each with sample sizes of 37 participants…” to “……each with **a** sample size**~~s~~** of 37 participants….”

On page 18, please rephrase this sentence "Among the 42 studies reviewed, there was a lack a general lack of rigor in the designed studies." to "Among the 42 studies reviewed, there was **~~a lack~~** a general lack of rigor in the designed studies."
