# Supplementary material for: Usual prevention in unusual settings: A scoping review of place-based health interventions in public-facing businesses
Source: PLoS One. 2025 Jan 24;20(1):e0317815. doi: 10.1371/journal.pone.0317815 (PMC11760570; doi:10.1371/journal.pone.0317815)
Supplement: Attachment — Submitted filename: Response Letter 12.30.24.docx [file pone.0317815.s007.docx]

December 29, 2024

Dear Dr. Chekuri,

Thank you for the opportunity to make some final corrections to the manuscript. Your comments were very helpful, and we have made each of the following revisions.

In the Abstract on Page 1, we have revised the sentence to now read: “The most common unconventional public-facing business settings for health interventions included barbershops or beauty salons…” We took out the number of studies because we agree it’s confusing and potentially misleading. You are also very correct that it is 34 (81%) of studies targeted Black populations. We have corrected this in the Abstract and in the Discussion, thank you very much.

On Page 2, we have revised the sentence as suggested to read: “Place-based interventions is one approach to reach people ‘where they are.’”

On Page 3, we have corrected a number of typos and reworded several sentences as you suggested. Thank you for these great suggestions.

On Page 5, we have taken out the word “reviewer.” On Page 7, we have corrected the wording to now read “…each with a sample size of 37 participants…”

On Page 18, we have corrected the sentence to now read “…there was a general lack of rigor in the designed studies." Thank you.
